# Exploring the relationship between trauma, mental health, and occupational performance in health science center students

**DOI:** 10.3389/fpubh.2024.1398238

**Published:** 2024-12-23

**Authors:** Feddah M. Ahmad, Dina M. Sajit

**Affiliations:** Occupational Therapy Department, College of Allied Health Sciences, Kuwait University, Kuwait City, Kuwait

**Keywords:** students, trauma, mental health, quality of life, occupational performance and participation

## Abstract

**Introduction:**

Attending university marks a pivotal yet stressful phase in students’ lives, characterized by significant adjustments to a new environment that can impact mental, emotional, and physical well-being. The journey through the acceptance and admissions process into university introduces substantial challenges, academic performance and changes to daily life. Such challenges and corresponding conditions can be intensified for students entering university with prior traumatic experiences.

**Objective:**

The purpose the study is: (a) to clarify the factors affecting student mental health by cataloging the prevalence and types of traumatic events (including PTSD and complex PTSD) that students experience, and (b) examine the relationship between students’ mental health and occupational performance through the assessment of satisfaction with daily activities and quality of life (QoL).

**Method:**

The research study utilized a descriptive cross-sectional design. Participants included students pursuing degrees in Medicine, Dentistry, Pharmacy, and Allied Health, (*N* = 223). Four self-administered questionnaires were employed: the International Trauma Questionnaire (ITQ), Beck’s Depression Inventory (BDI), the Self-Satisfaction of Daily Occupation (SDO), and World Health Organization Quality of Life Brief (WHOQoLBrief). Data was analyzed using descriptive statistics, and Spearman correlation.

**Result:**

Results from ITQ revealed participants experienced highest indicators of trauma were 68% affective dysregulation, and 65%, in avoidance and self-organization. Significant negative correlations were found between BDI scores and the quality-of-life social domain and quality of life environment domain (*p* = 0.001). Regarding BDI scores highest at 23.3% (*n* = 52) of students reported mild mood disturbance. SDO indicated that satisfaction levels increased with medium activity levels but decreased with high activity levels. In addition, a negative correlation was also found between SDO and BDI scores. Furthermore, a positive correlation between satisfaction with daily occupation (activity level) and WHOQoLBrief both physical, *p* > 0.001 and psychological, *p* = 0.006 was identified.

**Conclusion:**

This research investigates the cyclical impact of mental health on students’ daily activities, QoL, and occupational performance, particularly among those in Health Sciences Center. The study highlights how traumatic events and poor mental health contribute to dissatisfaction with daily tasks, which in turn leads to a decline in QoL and occupational performance emphasizing the importance of developing effective outreach strategies community.

## 1 Introduction

Students in medical and health-related programs often face significant mental and emotional challenges due to the rigorous demands of their studies. The transition to a medical campus introduces fluctuating expectations, heightened stress, and emotional vulnerability, particularly among students in medicine, dentistry, pharmacy, and allied health sciences (AHS), who are more prone to mental health issues like stress, anxiety, and depression compared to other disciplines (1, 2). Mental health disorders can result from past trauma and individual’s response to it can vary from person-to-person profound effects on students’ well-being (3). These traumatic experiences—unexpected events causing emotional distress—can further exacerbate these challenges, leading to lasting physical and psychological effects (4). These disorders can severely disrupt students’ daily activities, impairing their capacity to engage in academic work and extracurricular tasks.

Bodzaik et al. (3) reported trauma-induced mental health challenges contribute to decreased activity levels and reduced satisfaction with daily activities (i.e., academic, social, emotional, and physical) can negatively impact students’ well-being, and quality of life (QoL).

The impact on mental health is cyclical, as dissatisfaction with daily tasks due to poor mental health further contributes to the decline in QoL which directly affects their overall occupational performance (1). This study examines the occurrence of experienced traumatic events, prevalence of traumatic events, and students’ mental health trajectories impact on occupational performance in students at the Health Science Center at Kuwait University.

### 1.1 Literature review

Early adulthood marks the onset of many mental health issues, yet young adults often lack mental health literacy to understand diverse experiences (5, 6). Many of the diverse experiences occur without students recognizing or admitting what the DSM-5 categorizes as a “traumatic event.” The American Psychiatric Association defines the events as experiences of actual or threatened death, serious injury, or intimate violence, either directly experienced, as a witness, by proxy, or repeated extreme exposure (7). These traumatic events may include confrontation with war, natural disasters, serious illness, sudden loss, and other overwhelming events (8, 9). When these events lead to symptoms (i.e., intrusive memories, avoidance, negative changes in mood and thinking, and changes in emotional and physical reactions) they are characterized by and in accordance with the DSM-5 as post-traumatic stress disorder (PTSD). PTSD symptoms may manifest months or years after a traumatic event (10).

Bodzaik et al. identified a significant correlation between past trauma and current mental health issues (3). Following such experiences, students may exhibit co-occurring symptoms of depression, substance abuse, and anxiety disorders (7, 11). In multiple studies regarding the effects of childhood trauma on mental health and academic achievement, Larson et al. showed a significant risk of mental health disorders and poor academic achievement when exposed to childhood trauma (12). Complex PTSD, often resulting from prolonged trauma in early life, such as neglect or abuse, is characterized by symptoms including shame, feelings of ineffectiveness, threat, social withdrawal, and despair (13). Watt et al. (14) reported that individuals exposed to four or more adverse childhood events face a significantly higher risk of developing depression and anxiety in adulthood. Similarly, Brandt et al. found that traumatic experiences can lead to lifelong mental health complications, with social isolation worsening both mental and physical health (i.e., depression, schizophrenia, reduced quality of life, and increased morbidity and mortality) (15). Therefore, problems stemming from trauma directly affect students’ capacity to engage in daily activities, satisfaction levels, and their QoL.

Students pursuing competitive degrees often experience higher rates of mental health issues compared to the general population due to the pressures of academic and professional training, as well as financial burdens (16–18). Mental health challenges are particularly prevalent among health science students, who face the dual demands of excelling academically and clinically. These challenges can significantly affect students’ occupational performance, impacting their ability to engage in academic, social, and personal activities within the school environment, such as completing schoolwork, managing personal care, and interacting with peers (19, 20). Mental health issues, including stress, anxiety, and depression, play a critical role in shaping both academic performance and overall well-being (21).

Winter and Olivia revealed that a substantial number of health science students experience mental health challenges, with up to 85% reporting anxiety, 65% experiencing stress, and over 50% exhibiting symptoms of depression (22). These conditions negatively affect student learning, decision-making, and long-term mental and physical health. Additionally, trauma may place further strain on students’ mental health during university (9, 23). For example, the COVID-19 pandemic, as noted in Campbell et al. (23), significantly impacted mental well-being globally, exacerbating social isolation, depression, and anxiety, particularly among students (24). These mental health challenges may be closely linked to reduced occupational performance, defined as the ability to carry out meaningful daily activities, which is shaped by the interaction between an individual’s skills—physical, cognitive, and emotional—and their environment (20). According to Quek et al. (17) if medical students do not seek necessary support, they risk ongoing mental health issues throughout their careers, which can result in a decline in professionalism, academic performance, and empathy toward patients.

### 1.2 Objective

This study aims (a) to clarify the factors affecting student mental health by cataloging the prevalence and types of traumatic events (including PTSD and complex PTSD) that students experience, and (b) to examine the relationship between students’ mental health and occupational performance through the assessment of satisfaction with daily activities and quality of life (QoL).

## 2 Methodology

### 2.1 Study Design

A descriptive cross-sectional design was used because it is efficient, cost-effective, and provides a clear snapshot of a population’s health status at a single point in time, which is useful for recognizing public health needs (25). Additionally, it facilitates comparative analysis across different demographic groups and supports hypothesis generation for future research. As Wang and Cheng. and Thomphson et al. (26) reported this design is relevant for public health research assessing the prevalence of health-related behaviors and outcomes. None the less, every study design has its limitations. Cross-sectional studies are unable to establish causality due to the simultaneous measurement of exposure and outcome (27, 28). Incongruence, Maier et al. (27) and Mann (28) found descriptive cross-sectional designs prone to recall bias. Mainly because research is based on inaccurate reporting of past exposures or behaviors and that data collected at one point in time constricts a researcher’s ability to assess trends or long-term effects.

### 2.2 Sample

Participants included 240 students majoring in Medicine, Dentistry, Pharmacy, and Allied Health (N = 240). The self-administered questionnaire comprised four assessments: Self Satisfaction of Daily Occupation (SDO), International Trauma Questionnaire (ITQ), Beck’s Depression Inventory (BDI) and World Health Organization Quality of Life Brief (WHOQoLBrief).

Inclusion criteria: Students enrolled at Kuwait university in the faculties of Allied Health Science, Medicine, Pharmacy, and Dentistry. Students of all ethnic backgrounds and between 18 and 28 years. All assessments submitted need to have complete responses.

Exclusion criteria: Students not enrolled in the specified faculties at Kuwait University and individuals below 18 or above 28 years old. Incomplete survey or missing information from the survey will result in the participants being excluded from the study.

#### 2.2.1 Outcome assessment tool

The research study employed four self-administered questionnaires to gather data from the student body. Beck’s Depression Inventory (BDI-II) was utilized to assess depression levels among participants, as it has shown convergent validity (29, 30). Ghareeb (31) conducted a validation study of the Arabic BDI and found it to be reliable and valid for assessing depression in Arabic-speaking populations, with psychometric properties comparable to the original English version.

Several studies have validated the Arabic version of the BDI, demonstrating that it maintains high levels of internal consistency and validity (32). AlAnsari (33) reported a BDI Coefficient alphas were computed for samples of male and female undergraduates recruited from Palestine, Lebanon, Syria, Jordan, Saudi Arabia, Kuwait, Qatar, Bahrain, U.A. Emirates, Oman, Yemen, Egypt, Sudan, Tunisia, Libya, Algeria and Morocco with values of alpha ranged between 0.82 and 0.93.

The International Trauma Questionnaire (ITQ), available in both Arabic and English, served as a reliable measure of PTSD and Complex PTSD, providing diagnoses for symptoms such as avoidance, sense of current threat, and functional impairment. Nielsen et al. (34) have emphasized the cross-cultural validity of the ITQ, with the Arabic translation maintaining psychometric integrity.

Furthermore, the Satisfaction with Daily Occupations (SDO) scale, validated and reliable, encompassed nine items covering various occupational areas, including work, home management tasks, self-care, and leisure activities. Manee et al. (35) reported the SDO adapted and validated for use in Arabic-speaking populations. The researchers indicated the version comparable to the original version in both clinical and non-clinical settings and adapted to ensure cultural and linguistic inclusivity for the population. The study also indicated internal consistency was tested by Cronbach’s alpha analysis with SDO satisfaction scale demonstrated an overall raw alpha coefficient between 0.759 and 0.817 (33). In the study there were no challenges faced due the use of a translated version maintaining a high level of internal consistency, test–retest reliability, and validity.

Lastly, the World Health Organization Quality of Life Brief (WHOQOL-BREF) was utilized to assess participants’ overall quality of life, given its established validity and reliability (36). The WHOQOL-BREF assesses quality of life across four key domains: physical health, which includes mobility and energy; psychological health, covering emotional well-being and self-esteem; social relationships, focusing on personal connections and social support; and environment, involving safety, financial resources, and access to healthcare. These domains are interconnected, with each influencing the others (36). The assessment tool was selected based on their robust psychometric properties and accessibility in multiple languages to ensure inclusivity within the diverse student population. Ohaeri and Awadalla (37) conducted a psychometric evaluation of the Arabic WHOQOL-BREF among Arabic-speaking populations and found that it maintained high internal consistency, test–retest reliability, and construct validity. The internal consistency values for the full questionnaire and the domains had a Cronbach’s alpha ≥0.7. Other studies have supported these findings, confirming the instrument’s cross-cultural applicability (38, 39).

### 2.3 Ethical consideration

Ethical Approval was obtained from Health Science Center Ethical Committee at Kuwait University and Kuwait Ministry of Health.

### 2.4 Procedures

The target population for this study was approximately 4,000 students at Kuwait University in Jabriya, with a recruitment goal of N = 300 participants. The calculation of the sample size gave an approximation of 255 with a confidence level of 90%, a 5% margin of error. An approximation (non-probable) participant sample was increased due to geographical & virtual (users) proximity, availability in willingness to participate based on sensitivity of topic and aims. The research study included a survey. The survey was constructed using Google Forms and disseminated via various social platforms such as WhatsApp and Twitter. Students were invited to complete the survey during lectures by scanning a Quick Response (QR) code. Confidentiality and anonymity were rigorously maintained by a secure research laptop and a two-factor authentication system during data assessment. No names or contact details were collected, and participants were assigned numerical identifiers to replace personal information. This strategy effectively minimized bias and protected sensitive data, ensuring that the analysis remained focused on the collected information rather than the individuals.

Participants received consent forms detailing and outlining the study’s procedures, which they acknowledged by selecting “yes” to confirm participation. A demographic data form was incorporated into the survey to gather information on participants’ age, gender, educational level, GPA, and marital status. This comprehensive approach to recruitment and data collection aimed to ensure a diverse and representative sample for the study.

### 2.5 Data Collection

As the procedures were followed to utilize four self-administered standardized assessments were provided. Researchers were able to collect 240 participants responses, addressing various aspects of students’ psychosocial well-being and traumatic experiences on campus. While using the four assessments, the Beck’s Depression Inventory-II (BDI-II) was utilized as a screening tool for depression and psychosocial factors, known for its wide usage and effectiveness (40). The International Trauma Questionnaire (ITQ), served as a valid and reliable measure of PTSD and Complex PTSD, capturing characteristics such as avoidance, sense of current threat, negative self-concept, disturbances in relationships, and disturbances in self-organization impairment. It provides valuable insights into types of students’ traumatic experiences. The SDO scale comprises nine items covering various occupational domains, including work, home management tasks, self-care, and leisure activities. Lastly, to assess participants’ quality of life, the WHOQoLBrief assessment was administered and recognized for its validity and reliability (36). Al Maqbali et al. (38) confirmed the cross-cultural applicability and offering a comprehensive understanding of individuals’ overall well-being and capturing different facets of students’ experiences.

### 2.6 Data Analysis

Data analysis was conducted using SPSS-26. Descriptive statistics summarized age and GPA with means and standard deviations, while frequencies and percentages were used for gender, relationship status, and faculty. Spearman correlation assessed relationships between quality of life and depression (WHOQOL-Brief & BDI) and between satisfaction with daily occupations (SDO) and depression (BDI). Any missing data resulted in an immediate removal of the participant due to unmet inclusion criteria. Data transformations was applied to meet the assumptions of the statistical tests through converting and structuring data into usable format to be analyzed and match SPSS program for output.

## 3 Results

A total of 240 students participated in the study, representing various faculties. Most participants, constituted 88.8% of the sample after exclusions, were female (*n* = 198). This gender distribution is reflective of the higher proportion of female applicants admitted to medically based professions at Kuwait University. The faculty of Allied Health Sciences accounted for 98% of participants (*n* = 219), encompassing diverse majors such as physical therapy, occupational therapy, radiation sciences & nuclear medicine, medical laboratory sciences, and health informatics & information management.

Seventeen participants were excluded due to reasons such as lack of consent, incomplete responses, and sensitivity of topics. As per exclusion criteria incomplete assessments were not added into data analysis. The withdrawal of participants and exclusion of participants may have an impact on results either due to incomplete data ensures lack of representation of the target population and external validity. Therefore, a lack of diversity and complexity of the population introduces a bias. Due to the sensitivity of the research topic, many questions and responses may/would have been to invasive or induced feelings of exploitation.

Table 1 presents several demographic characteristics of participants in statistical data in percentages. Regarding marital status, 92% of participating students were single at the time of the study (*n* = 207). The demographic characteristics further revealed an average age of 20.83 years (SD: 1.712) and a mean GPA of 2.789 (SD: 0.336), providing insights into the profile of the study participants (see Table 1).

**Table 1:** Demographic characteristics of participants (*n* = 223).

	% (*n*)	m ± (SD)
Age		20.83 **±** (1.712)
GPA		2.789 **±** (0.336)
Gender		
Male	11.2 (25)	
Female	88.8 (198)	
Relationship status		
Single	92.8 (207)	
Married	6.3 (14)	
Divorced	0.9 (2)	
Faculty		
AHS	98.2 (219)	
Pharmacy	0.9 (2)	
Medicine	0.9 (2)	
Dentistry	0 (0)	

The study only indicated the prevalence of traumatic events but does not convey a correlation of trauma and mental health or occupational performance. Other research indicates diverse activity affecting occupational performance. These challenges can significantly impact students’ ability to engage in academic, social, and personal activities within the school environment, such as completing schoolwork, managing personal care, and interacting with peers (19, 20). Different types of activity and correlations based on specific PTSD symptoms were not researched but an implication for future investigation. Furthermore, research on occupational performance (i.e., based on both academic vs. non-academic activity) and types of mental health challenges is also another avenue for research.

**Figure 1:**
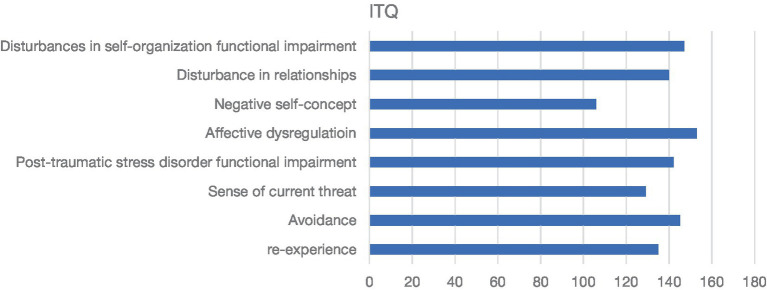
International Trauma Questionnaire (ITQ) eight domains and common distribution diagnosis.

Figure 1 shows the International Trauma Questionnaire’s eight domains in students with PTSD and complex PTSD at Kuwait University. The International Trauma Questionnaire’s presented a majority in 68% (*n* = 153) effective dysregulation. The prevalence for the other domains were 60% (*n* = 135) re-experiencing, 65% (*n* = 145) avoidance, 57% (*n* = 129) sense of current threat, 63% (*n* = 142) PTSD functional impairment, 48% (*n* = 109) negative self-concept, 62% (*n* = 140) disturbance in relationships, and 65% (*n* = 147) disturbance in self-organization functional impairment.

**Figure 2:**
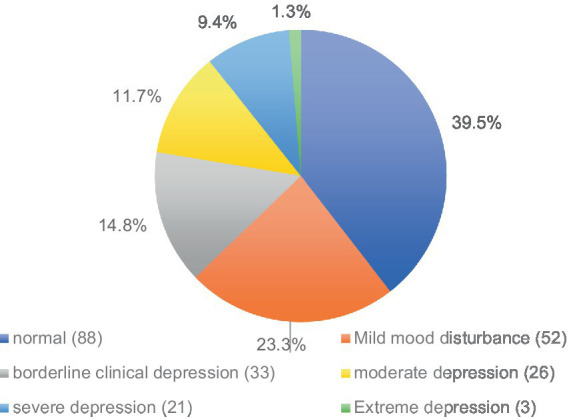
Becks depression inventory-II (BDI) population’s depression levels (*n*)/%.

The Beck’s Depression inventory-II (BDI-II) was utilized to evaluate psychosocial aspects, specifically focusing on depression among students. Figure 2 illustrates the distribution of depression severity levels among participants. Notably, (39.5%, *n* = 88) of participants exhibited no signs of depression, while (23.3% *n* = 52) experienced mild mood disturbances. A smaller proportion, accounting for (1.3% *n* = 3) of participants, reported experiencing extreme depression. The remaining participants (37.2%, *n* = 80) were distributed across categories indicating borderline clinical depression (14.8%, *n* = 33), moderate depression (11.7%, *n* = 26), or severe forms of depression (9.4% *n* = 21), highlighting the varying degrees of depressive symptoms observed within the sample.

Table 2 below, presents Spearman correlation coefficients between depression and quality of life (QOL) domains. Depression shows negative correlations with QOL Social (−0.225) and QOL Environment (−0.278), indicating lower QOL as depression increases. Both correlations are statistically significant at the 0.05 and 0.01 levels. Additionally, a positive correlation exists between QOL Social and QOL Environment, with a highly significant *p*-value of 0.001. These findings underscore the impact of depression on various aspects of QOL among students.

**Table 2:** Correlation of BDI with WHOQoLBrief.

	BDI	QOLE	QOLS
BDI	–	−0.278^**^	−0.225^**^
QOLE	–	–	0.576^**^
QOLS	–	–	–

*Correlation is significant at the 0.005 level (2-tailed). **Correlation is significant at the 0.001 level (2-tailed). The *p* value (0.001) was statically significant with QOLE, QOLS and depression. QOLE: quality of life environment; QOLS: Quality of life social.

As results indicated that when assessing QoL in terms of physical, psychological, social, and environmental domains, these aspects have a positive correlation with daily activities, both in terms of activity levels and satisfaction. Therefore, overall occupational performance is significantly influenced by mental health, as it directly affects satisfaction with daily activities and quality of life.

In Table 3, presents Spearman correlation coefficients with a negative correlation is shown between BDI and Satisfaction with Daily Occupation Activity Level (SDOA) and Satisfaction with Daily Occupation Satisfaction Level (SDOS); however, a positive correlation existed between SDOA and SDOS.

**Table 3:** Correlation between daily occupation (Activity level and satisfaction) AND BDI.

	BDI	SDOA	SDOS
BDI		−0.300**	−0.120
SDOA			0.270**
SDOS			

**Correlation is significant at the 0.001 level (2-tailed). BDI: becks depression inventory. SDOA: satisfaction with daily occupation activity level. SDOS: satisfaction with daily occupation satisfaction level.

**Figure 3:**
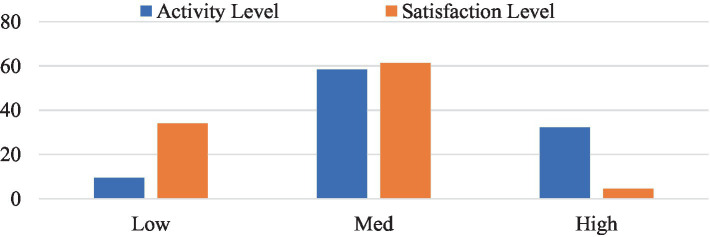
Satisfaction with daily occupation (%).

In Figure 3, below represents percentages between activity level and satisfaction levels. The figure demonstrates that when activity level is high the satisfaction level is low whereas when activity level is medium the satisfaction level increases.

While in Table 4 presents Spearman correlation coefficients with a positive correlation between satisfaction with daily occupation (activity level) and QOL both physical, (*p* > 0.001) and psychological, *p* = 0.006. The found result indicates that activity levels were based on a sense of belonging for motivation and stress buffering. While the positive correlation between satisfaction with daily occupation (satisfaction level) indicates certainty about belonging improves academic and social integration. Furthermore, (Table 4) indicated that there is a correlation between satisfaction with daily activities (satisfaction level) and QOL Social, *p* = 0.001 and Environment, *p* > 0.001.

**Table 4:** SDO (Activity AND satisfaction levels) relationship with QOL.

	SDOA	SDOS	QOLPhy	QOLPsy	QOLS	QOLE
SDOA	1.000	0.339^**^	**0.184** ^**^	**0.204** ^**^	0.117	0.115
		0.000	**0.006**	**0.002**	0.082	0.085
		223	223	223	223	223
SDOS	–	1.000	**0.214** ^**^	**0.249** ^**^	**0.231** ^**^	**0.314** ^**^
			**0.001**	**0.000**	**0.001**	**0.000**
			223	223	223	223

*Correlation is significant at the 0.005 level (2-tailed). **Correlation is significant at the 0.001 level (2-tailed). The *p* value (0.001) was statically significant.

## 4 Discussion

The study cataloged various types of traumatic events, including physical and emotional abuse, accidents, natural disasters, and experiences of violence. The data showed that approximately 68% (153) of students reported exposure to traumatic event resulting in effective dysregulation. A notable 63% (142) exhibited signs of PTSD, and complex PTSD symptoms. These findings align with previous research that connects exposure to trauma with increased risks of anxiety, stress, and depression, further reinforcing the association between trauma and deteriorating mental health (5, 12). According to Cross et al. (41) explained that chronic stress could promote adverse epigenetic and neurobiological mechanisms, and the timing of trauma impacts neurobiological processes. For instance, interpersonal trauma involves emotional abuse, emotional neglect, physical abuse, physical neglect, sexual abuse, and witnessing violence leading to chronic stress (5). Therefore, these clients are at risk for negative cognitive, emotional, and psychiatric outcomes. They indicated students who experienced emotional abuse tended to report higher levels of anxiety and depression, which more severely impacted their psychological and social QoL domains.

Bodzaik et al. traumatic experiences often lead to long-term emotional distress and psychological impairment, which significantly impact one’s mental health (3). The study highlights those different types of traumas prove to be connected to mental health challenges presented in a decline and dissatisfaction in occupational performance in varied ways. On the other hand, students who had experienced physical trauma were more likely to report disruptions in their physical QoL domain. These nuances in trauma types offer critical insights into how tailored interventions may address the specific mental health needs of students and improve their overall well-being. Moreover, Middle Eastern medical students tend to face increased stress due to high academic demands, cultural expectations, and reluctance to seek mental health support due to preexisting stigma regarding mental health (42–44).

Participants were predominantly female (88.8%), consistent with gender norms in Middle Eastern regions (45, 46). Furthermore, in many Middle Eastern societies, medicine is seen as an extension of nurturing roles traditionally assigned to women, making it a socially acceptable field for women to pursue, moreover women tend to make up more than half of medical graduates in Kuwait (47). This alignment between cultural expectations and professional paths explains why a higher percentage of women are entering medicine compared to other professions and why most participants in this study were females. Also finding that gender was not a statistically significant moderator on the prevalence of anxiety among medical students in the Middle East and the traditional notion of ‘girls are worriers and boys are carefree and worry-free’ may not hold true in the context of a group of medical students. However, studies suggest psychological distress may be higher among female students in the United States and Canada (2)

To increase QoL in students, enhancing satisfaction is suggested to occur through environmental influence on social participation to decrease mental health challenges (48). Data from the BDI and WHOQOL-BREF indicated negative correlations between QoL and depression and a positive correlation between QoL and satisfaction. High activity levels were associated with low satisfaction (Figure 2), possibly due to overwork, necessitating further investigation into causes of depression among students. The inverse relationship between QoL and depression can be attributed to the fact that depression often diminishes the ability to find enjoyment in life, reduces motivation, and limits engagement in meaningful activities, all of which negatively affect well-being (49). On the other hand, the positive link between QoL and satisfaction suggests that engaging in fulfilling activities and experiencing personal contentment are essential for emotional and psychological health (50). As mental health deteriorates, so does occupational performance, indicating that interventions targeting mental health issues can have a significant positive impact on QoL (49).

Furthermore, high levels of activity are often linked to lower satisfaction, particularly in environments where people face excessive demands and time pressures, may lead to burnout, fatigue, reduced overall well-being and life satisfaction. Sonnentag and Fritz (51) reported continuous engagement in high-paced tasks without adequate recovery time, negatively impacts emotional and physical health, contributing to feelings of dissatisfaction. This highlights the importance of balancing activity levels with personal fulfillment to enhance overall QoL.

The negative relationships between depression and levels of activity and satisfaction (BDI with SDOA and SDOS), suggested depressed individuals may engage less with their environment maybe influenced by several key factors (i.e., reduced motivation and energy, leading to lower engagement in physical and social activities). Gotlib and Hammen (52) found this lack of participation can further result in diminished life satisfaction.

To assess the broader impact of mental health on students’ occupational performance, this study utilized the SDO and WHOQoL-Brief outcome measures. The SDO measure helped to gauge students’ contentment with their academic and non-academic activities, while the WHOQoL-Brief provided insight into the four main domains of quality of life: physical, psychological, social, and environmental well-being. Both measures demonstrated that mental health issues, particularly those linked to trauma, significantly detract from students’ satisfaction with their daily activities and overall well-being. Research confirms that poor mental health adversely affects academic performance, including students’ concentration, retention, and overall engagement (12). Similarly, mental health disturbances negatively affect non-academic activities such as social participation, personal care, and leisure, all of which are crucial components of QoL and occupational performance (41).

More so, social withdrawal is another critical factor; those experiencing depression may isolate themselves, further reducing their satisfaction due to a lack of social interactions and support creating a feedback loop where low levels of activity and satisfaction exacerbate depressive symptoms (52). Therefore, factors such as social support, goal setting and achievement, mindfulness, relaxation techniques and physical activity can counteract these negative impacts of depression on activity levels and satisfaction (53). Engaging in activities that individuals find meaningful, leisure, or hobbies, has a significant positive effect on their overall well-being. Eklund and Backstrom (54) highlight that people who are content with their daily activities tend to experience greater life satisfaction, emotional health, and a sense of purpose. Moreso, they indicate uncertainty about belonging impedes academic and social integration (55). The results show that QoL, measured across physical, psychological, social, and environmental domains, positively correlates with students’ satisfaction and engagement in daily activities. Mental health challenges, particularly those stemming from trauma, affect both academic performance and non-academic activities. Students experiencing depression, for instance, often struggle with concentration and academic engagement, leading to lower performance. These mental health issues also affect non-academic tasks like self-care and social interactions, all of which are essential for maintaining a balanced and fulfilling life (56). They also found regular participation in fulfilling tasks helps to build emotional resilience and alleviate stress (57). Therefore, finding satisfaction in daily occupations is a key factor in enhancing quality of life, emphasizing the importance of maintaining a healthy balance in everyday activities.

## 5 Conclusion

As the research study illuminated, many individuals experiencing PTSD and depression may not recognize the profound impact of these conditions on their overall well-being, which is often reflected in a diminished quality of life (QoL) across various domains, including emotional, social, and physical functioning in turn effecting occupational performance. This study aimed to clarify the intricate relationship between mental health and, occupational performance among students. The findings underscore that trauma-induced mental health problems are a significant barrier to student satisfaction with daily activities and overall well-being. The study described that depression is significantly associated with lower QoL, as individuals often experience impaired functioning in multiple areas of life, irrespective of their activity and does not necessarily translate to personal satisfaction or happiness. Studies have shown that quality, rather than quantity, in daily activities fosters greater well-being, as meaningful engagement in fewer activities often enhances life satisfaction and reduces feelings of burnout (58).

Bodzaik et al. research pushed in preventing adversity, creating awareness and recognition, and strengthening resilience are strategies he suggests addressing mental health disorders related to trauma (3). In addition, counseling, reconciliation programs, an introduction of policies related to common risk factors, and early intervention strategies within communities will provide more positive public mental health outcomes (59). In conclusion, by addressing these mental health challenges through appropriate interventions, we can positively influence students’ occupational performance both through satisfaction of daily activities and quality of life, thereby promoting better academic and personal success.

### 5.1 Limitations

The research study presents several constraints and limitations that may have affected the interpretation, generalizability, and validity of its findings. These limitations clarify the scope of the research and involve factors such as gender bias, lack of diversity in faculty, major, and relationship status, as well as the use of lengthy, time-consuming survey questions. Self-reported questionnaires, although widely used, carry inherent limitations. They are prone to response biases like social desirability, inaccurate memory recall, and varying interpretations of questions by individuals. Additionally, they may not capture the full complexity of behaviors or emotions, with fixed response options restricting nuanced expression. Issues like self-perception, sample bias, and timing effects can further compromise data quality. The reliance on self-reported information introduces the risk of exaggerated, dishonest, or incomplete responses. While tools like the BDI-II and ITQ are robust, acknowledging potential biases—such as social desirability or mood influences—offers a more critical view of the methodology.

Moreover, the cross-sectional design is vulnerable to recall bias and misclassification, as participants may inaccurately report past exposures or behaviors. The convenience sampling method may also have excluded certain demographic groups, and students more engaged with social media or lectures may be overrepresented in the sample. Researchers attempted to mitigate this by targeting students during class lectures and breaks to reach less interested or engaged individuals, but this effort had its limitations. The study’s design also prevents establishing cause-and-effect relationships, as data was collected at a single point in time, limiting insight into long-term effects. Additionally, the lack of male participants introduced gender bias. This could be explained by the fact that a majority of students in allied health sciences, medicine, pharmacy, and dentistry at Kuwait University are female (44). However, this overrepresentation of female participants may affect the findings, as mental health issues can manifest differently between genders.

### 5.2 Implications

This research highlights the urgent need for universities, particularly in health-related fields, to address the complex stressors affecting students’ mental health and overall well-being. To reduce stress-related incidents affecting the physical, social, and mental well-being of the university community, academic expectations and the environment must be better aligned. On-campus recreational outlets specifically designated for allied health science students (medical campus) are needed. Occupational therapy can inform the development of targeted interventions and support systems tailored to the unique needs of students in medical and health-related fields. Potential interventions include workshops on coping strategies and motivational speakers who address stress and lifestyle from religious, social, and cultural perspectives Walton & Cohen (60).

Further research is necessary to understand the impact of traumatic experiences and depression on students in these fields. Such insights could lead to improvements in students’ quality of life (QOL). Student stress arises from multiple sources, including academic pressure, social relationships, time management, and family expectations, while other contributing factors often go overlooked, highlighting the need for more research in this area. Tailored interventions, such as counseling services and stress management programs, have been shown to enhance mental health outcomes and, consequently, QOL in this population (61). By addressing these issues, this study underscores the need for customized support services that directly address the mental health needs of allied health science students, ultimately contributing to their academic success and personal well-being.

Additionally, recognizing the distinct yet interconnected nature of stress, anxiety, and depression is crucial for fostering a more resilient and mentally healthy academic community. Addressing the mental health needs of medical students is crucial, as untreated trauma and stress can lead to long-term consequences, including professional burnout and compromised patient care (2). Future research on coping mechanisms and the long-term effects of traumatic experiences will be instrumental in shaping policies and support systems that better serve students’ academic and occupational performance.

## Data Availability

The original contributions presented in the study are included in the article/supplementary material, further inquiries can be directed to the corresponding author.
